# Practice variability in the management of critical pertussis: a multicenter survey of pediatric intensivists in the Arabian Gulf Cooperation Council region

**DOI:** 10.3389/fped.2026.1662218

**Published:** 2026-03-06

**Authors:** Mohammad Alghounaim, Mohamad-Hani Temsah, Abdulrahman Aldaithan, Manu S. Sundaram, Amal Al Daylami, Musaab Ramsi, Saif Awlad Thani, Yasser Kazzaz, Abdulla Alfraij

**Affiliations:** 1 Pediatric Department, Amiri Hospital, Kuwait City, Kuwait; 2 Pediatric Intensive Care Unit, Pediatric Department, College of Medicine, King Saud University, Riyadh, Saudi Arabia; 3 Evidence-Based Healthcare and Knowledge Translation Research Chair, Family Medicine Department, College of Medicine, King Saud University, Riyadh, Saudi Arabia; 4 Pediatric Intensive Care Unit, Pediatric Department, Farwaniya Hospital, Sabah Alnasser Area, Kuwait; 5 Pediatric Intensive Care Unit, Pediatrics Division, Ahmadi Hospital, Kuwait Oil Company (KOC), Ahmadi, Kuwait; 6 Pediatric Intensive Care Unit, Sidra Medicine, Doha, Qatar; 7 Department of Pediatrics, Weill Cornell Medicine, Doha, Qatar; 8 Pediatric Intensive Care Unit, Government Hospital, Manamah, Bahrain; 9 Pediatric Critical Care Unit, Sheikh Khalifa Medical City (SKMC), Abu Dhabi, United Arab Emirates; 10 Pediatric Intensive Care Unit, The Royal Hospital, Muscat, Oman; 11 Department of Pediatrics, Ministry of National Guard Health Affairs, Riyadh, Saudi Arabia; 12 College of Medicine, King Saud Bin Abdulaziz University for Health Sciences, Riyadh, Saudi Arabia; 13 King Abdullah International Medical Research Center, Riyadh, Saudi Arabia

**Keywords:** pertussis, pediatric intensive care, leukoreduction, hyperleukocytosis, physician knowledge

## Abstract

**Background:**

Critical pertussis continues to cause significant morbidity and mortality in infants necessitating pediatric intensive care. Despite advances in supportive care, knowledge gaps persist. This study aimed to examine institutional capacity, physician knowledge, and practice variability in managing critical pertussis among pediatric intensive care units (PICUs) across the Gulf Cooperation Council (GCC) countries.

**Methods:**

A cross-sectional internet-based survey was distributed to PICU physicians across the six GCC countries between December 1, 2024, and January 31, 2025. Demographic information, clinical experience, diagnostic resources, and therapeutic approaches were collected. A multivariable generalized linear regression (Gamma) model identified factors associated with pertussis knowledge scores.

**Results:**

Among 185 respondents, almost 70% of participants were male, 62.7% were specialists or consultants, and around half (47%) were certified pediatric intensivists. Access to mechanical ventilation was almost universal (98.4%), yet extracorporeal membrane oxygenation was available in only 24.3% of centers. Polymerase chain reaction-based diagnosis was widely available, but more than one-third (36.2%) of participants reported a test turn-around-time of at least two days. A majority (66%) of physicians used exchange transfusion for hyperleukocytosis, but white blood cell thresholds varied widely. Institutional protocols were lacking in over 40% of centers. The average pertussis knowledge score was 9.52 out of 13 questions (SD ±1.72). Physician's clinical experience showed a strong and graded association with pertussis knowledge.

**Conclusions:**

This study highlights the heterogeneity in pertussis management practices across the GCC PICUs, compounded by variability in resources and different institutional guidelines. Findings highlight the urgent need for standardized protocols to harmonize pertussis care.

## Key messages

This multinational study of pediatric intensive care physicians revealed inconsistent critical pertussis management: variable leukoreduction thresholds (30–70 × 10^9^/L), limited ECMO access (24%), and diagnostic delays (≥2-day PCR turn-around in 36%). Despite moderate-to-high knowledge (mean score 9.52/13), gaps persisted in transmission awareness. Clinical experience predicted knowledge. Standardized guidelines are needed to harmonize care.

## Introduction

Pertussis, caused primarily by *Bordetella pertussis*, presents with variable clinical features, with infants often experiencing a more severe form of the disease. In this population, pertussis can lead to life-threatening complications, largely due to their immature immune systems and incomplete vaccination status (1). Despite advances in global immunization efforts, pertussis continues to cause serious morbidity requiring pediatric intensive care unit (PICU) admission, particularly in infants under three months of age (2). After limited pertussis circulation during the coronavirus disease 2019 (COVID-19) pandemic, there has been a sharp increase in pertussis cases globally between 2023 and 2024 (3–5). The Gulf Cooperation Council (GCC) countries, which historically maintained robust childhood vaccination programs, have observed similar epidemiologic trends, including increased hospitalization and PICU admissions (6–8). Although published epidemiologic data from the broader Middle East and North Africa (MENA) region are limited, pertussis continues to pose a substantial burden, with a similar post-COVID-19 resurgence (9–11).

The treatment strategy for pertussis relies heavily on the use of macrolide antibiotics to limit transmission and modify the disease course. However, the effectiveness of macrolides in relieving pertussis-related symptoms and complications beyond the prodromal phase of the disease is questionable (5). Although most cases of pertussis follow a relatively benign clinical course, around 10%–25% of hospitalized infants receive care in PICUs (12, 13). Severe or critical disease is more likely to occur in young infants—particularly those younger than 3 months—or in those with prematurity, low birth weight, incomplete vaccination, marked leukocytosis, or pulmonary hypertension (6, 14). Critical pertussis is defined as pertussis disease that results in PICU admission. Malignant pertussis is a rare subtype of critical pertussis characterized by hyperleukocytosis, pulmonary hypertension, and cardiovascular collapse (15). Critical and malignant pertussis pose many management challenges in intensive care settings (16). Therapeutic modalities such as leukoreduction therapies (e.g., exchange transfusion, leukapheresis) and pulmonary vasodilators are used inconsistently due to the lack of standardized protocols and limited resources (17).

Despite increasing global attention to the resurgence of pertussis, much of the research, both within the GCC and internationally, has centered on epidemiological trends, with comparatively less emphasis on clinical management strategies in critical care settings. The limited availability of region-specific data, along with the broader lack of consensus on interventions such as leukoreduction thresholds and escalation protocols, highlights the importance of understanding current clinical decision-making practices across diverse healthcare systems. We hypothesized that significant variability exists across GCC countries in institutional preparedness, physician decision-making, and therapeutic interventions for critical pertussis care. This study aimed to explore institutional capacities, physicians' knowledge, and practice variability in the management of critical pertussis among PICUs across all GCC countries.

## Methods

### Study design and participants

This multicenter cross-sectional study employed an internet-based survey to evaluate institutional preparedness, clinical practices, and physician knowledge related to critical pertussis management in PICUs across the six GCC countries: Bahrain, Kingdom of Saudi Arabia, Kuwait, Oman, Qatar, and United Arab Emirates. Data were collected between December 1st, 2024 and January 31st, 2025. The targeted population included pediatric intensivists and pediatricians working primarily in a PICU. Physicians not practicing primarily in a PICU or those working outside the GCC region were excluded. The survey was distributed through professional networks and institutional contacts and responses were collected electronically using Survey Monkey (Survey Monkey, San Mateo, California, USA). Three reminders were sent during the study period.

### Survey instrument (Supplementary Appendix A)

A structured 50-item questionnaire was developed based on a literature review and existing clinical guidelines related to pertussis management (17–19). It consisted of four main domains: (1) demographic and professional background; (2) institutional capacity and available diagnostic tools; (3) clinical management practices for pertussis, including critical care protocols; and (4) knowledge assessment based on 13 true-false questions covering disease transmission, clinical presentation, prevention, and treatment strategies. The questionnaire was piloted and validated by a panel of six experts, including pediatric intensivists and infectious disease specialists.

Participation was voluntary, and the informed consent statement was embedded at the beginning of the survey. No identifiable personal data were collected, ensuring full anonymity. Duplicate entries were avoided by restricting one response per device.

### Statistical analysis

Descriptive statistics were used to summarize the data. Mean and standard deviation were applied to continuous variables, while frequencies and percentages were used for the categorically measured variables. Multiple response dichotomy analysis was used to describe variables with more than one selectable option. The Kolmogorov–Smirnov test was used to assess the normality of continuous variables, supported by histogram inspection. Physician knowledge scores were calculated as the proportion of correct responses (range: 0–13) and treated as a continuous outcome. Multivariable generalized linear regression analysis with gamma was applied to assess the statistically significant predictors for the physicians' total pertussis knowledge score, and the association between the tested predictor independent variables with the analyzed outcome dependent variables was expressed as an exponentiated beta coefficients (risk rate) with its 95% confidence intervals. Statistical significance was defined as a *p*-value <0.05. All analyses were conducted using SPSS software (IBM SPSS Statistics for Windows, Version 28.0).

### Ethical consideration

Ethical approval was obtained from the Institutional Review Board at Ahmadi Hospital in Kuwait (ref 9-2024).

## Results

One hundred and eighty-five PICU physicians had enrolled themselves into the study and completed the study questionnaire. Almost 70% of participants were male, 62.7% were specialists or consultants, and around half (47%) were board-certified pediatric intensivists (Table 1). The physicians' clinical experience was distributed as shown in Table 1. Most participants (73.6%) reside in either Kuwait or the Kingdom of Saudi Arabia.

**Table 1:** Physicians’ sociodemographic characteristics and working and professional-related factors (*N* = 185).

Variable	*n* (%)
Gender	
Male	129 (69.7)
Professional designation	
Medical Resident/Registrar	46 (24.9)
Fellow/Senior Registrar	23 (12.4)
Consultant/Specialist	116 (62.7)
Subspeciality	
Pediatric Intensivist (Board-Certified)	87 (47)
Pediatrician (working in PICU)	98 (53)
Type of healthcare facility	
Governmental Hospital	167 (90.3)
Private/other non-governmental Hospital	18 (9.7)
PICU size	
Small Capacity (≤10 beds)	46 (24.9)
Medium Capacity (11–20 beds)	69 (37.3)
Large Capacity (21–40 beds)	70 (37.8)
Available PICU-related resources	
Basic resources (e.g., Non-invasive ventilator, no Mechanical ventilators)	3 (1.6)
Moderate resources (e.g., Mechanical ventilators, No ECMO)	137 (74.1)
High-level resources (e.g., ECMO and other advanced critical care technologies)	45 (24.3)
Physician's years of experience	
≤5 years	33 (17.8)
6–10 years	53 (28.6)
11–15 years	42 (22.7)
≥16 years	57 (30.8)
Country of residence	
Bahrain	2 (1.1)
Kingdom of Saudi Arabia	58 (31.4)
Kuwait	78 (42.2)
Oman	17 (9.2)
Qatar	18 (9.7)
United Arab Emirates	12 (6.5)

ECMO, extracorporeal membrane oxygenation; PICU, pediatric intensive care unit.

Most physicians worked in a governmental hospital, consistent with the structure of healthcare systems across the GCC region. Furthermore, 24.9%, 37.3% and 37.8% of physicians worked in a small, medium, and large-capacity PICU, respectively. Among these, 74.1% reported working in ICUs with moderate resources (e.g., mechanical ventilation but no ECMO), while 24.3% had access to ECMO and other advanced technologies. Number of pertussis cases managed in the ICU varied among participants. Around 7% reported managing 11–20 cases during the past 12 months (Table 2). Although PCR tests are available to 98.4% of participants, 36.2% reported a prolonged test turn-around time (≥2 days), and 10.3% reported delays of 5 days or more. Additionally, only 21.6% performed PCR tests routinely on all patients admitted to PICU with respiratory symptoms. Furthermore, echocardiogram was reported to be performed routinely in critical pertussis patients among 37.8% of physicians. The primary trigger for suspecting malignant pertussis varied, with more than half of respondents (54.6%) citing hyperleukocytosis as the main indicator.

**Table 2:** Physicians’ perceptions about initial diagnostics and screening practices at their workplace.

Survey question	Frequency *n* (%)
How many critical pertussis cases admitted to the PICU/HDU have you personally managed in the last 12 months?	
No cases	13 (7)
1–2 cases	45 (24.3)
3–5 cases	65 (35.1)
6–10 cases	48 (25.9)
11–20 or more cases	14 (7.6)
What diagnostic tests are available for pertussis in your center?	
PCR (part of an extended respiratory panel)	104 (56.5)
PCR specific to pertussis	118 (64.1)
Serology	23 (12.5)
Culture	24 (13)
Not sure	6 (3.3)
What is the average turn-around time (time from sample collection to report) for PCR-pertussis testing at your center?	
Test not available	3 (1.6)
Hours (Up to 24 h)	115 (62.2)
Days (2–4 days)	48 (25.9)
≥5 day	19 (10.3)
Frequency of pertussis testing among patients with respiratory symptoms admitted to PICU	
Not sure	20 (10.8)
Routine	40 (21.6)
0–10%	48 (25.9)
11–20%	41 (22.2)
21%–50%	36 (19.5)
How frequently do you perform echocardiograms for pertussis cases admitted to your PICU?	
Never	4 (2.2)
In selected cases/situations	111 (60)
Always	70 (37.8)
Which one of the following is most likely to trigger you to suspect “Malignant Pertussis"?	
No sure/I don't know	1 (0.5)
Cardiovascular collapse	3 (1.6)
Hyperleukocytosis	101 (54.6)
Hypoxemia unresponsive to oxygen therapy	21 (11.4)
Pulmonary hypertension	21 (11.4)
Rapid clinical deterioration	35 (18.9)
Other (please specify)	3 (1.6)

HDU, high-dependency unit; PCR, polymerase chain reaction; PICU, pediatric intensive care unit.

**Figure 1:**
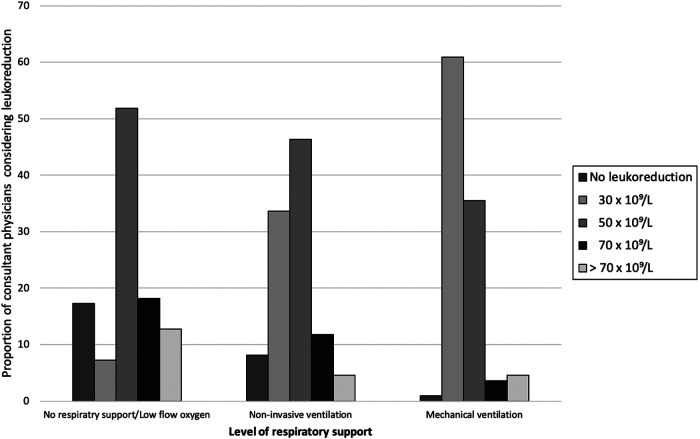
Proportion of reported white blood cell (WBC) threshold by PICU consultant for initiating leukoreduction categorized by the level of respiratory support.

Table 3 summarizes physicians’ overall approaches to managing hyperleukocytosis in critical pertussis, encompassing both supportive and leukoreductive strategies. Approximately two-thirds reported using exchange transfusion (68.6%) or hyperhydration (61.6%) as part of their management approach. One-and-half maintenance fluid therapy was the most commonly used fluid management rate in patients with hyperleukocytosis without pediatric acute respiratory distress syndrome (PARDS). The data demonstrated a trend for a more aggressive approach towards leukoreduction at lower white blood cell (WBC) counts in patients with greater respiratory compromise. Most physicians considered a WBC count of 50 × 10^9^/L for patients without oxygen requirement or with low-flow oxygen. However, once the patient is put on a mechanical ventilator, the majority of physicians may initiate leukoreduction therapy with counts 30 × 10^9^/L or more (Figure 1). When asked specifically about the primary modality used for leukoreduction, exchange transfusion (60.5%) was most commonly selected, followed by leukapheresis (24.9%). Among physicians who utilized a leukoreduction procedure, 69.4% believe it resulted in sustained clinical improvement in patients. Almost 70% of respondents identified intensivists performs leukoreduction, while 20% of them were done by hematologists. Moreover, 55.6% indicated that their institutions lack a formal policy or protocol for leukoreduction. Several challenges were identified in performing exchange transfusion, most commonly: risk of complications (54.9%) followed by lack of established guidelines (40.5%). Hemodynamic instability, and electrolyte imbalance were the two most perceived leukoreduction adverse effects.

**Table 3:** Physicians’ perceptions of pertussis management and related challenges.

Survey question	*n* (%)
Do you have a protocol for managing hyperleukocytosis in pertussis cases?	
No/not aware of any	103 (55.6)
Yes (rarely followed)	15 (8.1)
Yes (well-established)	67 (36.2)
What is your approach to managing hyperleukocytosis in critical pertussis? (select all that apply)	
Leukapheresis	58 (31.4)
Exchange transfusion	127 (68.6)
Hyperhydration (≥1.5x maintenance fluids)	114 (61.6)
Hydroxyurea	14 (7.6)
None of the above treatments/management	4 (2.2)
Who typically/mostly performs leukoreduction/exchange transfusion procedures at your facility?	
Unsure	5 (2.7)
Hematology team	37 (20)
PICU team	128 (69.2)
Other teams	15 (8.1)
From your experience, which leukoreduction methods do you primarily use for managing hyperleukocytosis in pertussis?	
Unsure/I have not used leukoreduction strategies	23 (12.4)
Leukapheresis	46 (24.9)
Exchange Transfusion	112 (60.5)
Cytoreductive medications (e.g., hydroxyurea)	1 (0.5)
Other	3 (1.6)
Which of the following have you used in the management of pulmonary hypertension in malignant pertussis?	
Inhaled Nitric Oxide (iNO)	117 (63.2)
Sildenafil	67 (36.2)
Milrinone	57 (30.8)
Heparin	6 (3.2)
ECMO	28 (15.1)
I have no experience in such a case	50 (27)
How often did you use ECMO in critical pertussis cases?	
Never used ECMO for pertussis	80 (43.2)
ECMO service not available in my center	73 (39.5)
1–5% of the cases	29 (15.7)
6–10% of the cases	3 (1.6)
In your clinical experience, how effective is ECMO in improving the outcomes of malignant pertussis?	
No experience with such cases	133 (71.9)
Ineffective	11 (5.9)
Minimally effective	14 (7.6)
Moderately effective	13 (7)
Highly effective	14 (7.6)
What challenges do you encounter when using exchange transfusion for critical pertussis? (select all that apply)	
Resource availability	32 (18.5)
Risk of complications	95 (54.9)
Lack of experience	37 (21.4)
Lack of guidelines	70 (40.5)
Vascular access	51 (29.5)
Uncertainty about its efficacy in pertussis	36 (20.8)
I have never used it before	20 (11.6)
What was the most common pertussis/clinical outcome you observed in patients who underwent Leukoreduction procedures like exchange transfusion or leukapheresis (not taking into account the effect on leukocyte count)?	
I have not used any leukoreduction procedure in my practice	41 (22.2)
No significant change in outcomes	19 (10.3)
Increased complications or worsening condition	4 (2.2)
Sustained clinical improvement	100 (54.1)
Temporary improvement followed by deterioration	21 (11.4)
What complications have you encountered with leukoreduction procedures?	
Hemodynamic instability	74 (68.5)
Electrolyte imbalances	49 (45.4)
Central venous catheter occlusion/malfunction	32 (29.6)
Secondary infection	15 (13.9)
Seizures	10 (9.3)
ARDS	31 (28.7)
Other	3 (2.8)

Regarding the management of pulmonary hypertension in malignant pertussis, 63.2% of physicians reported using inhaled nitric oxide (iNO), 36.2% used sildenafil, and 30.8% used milrinone. ECMO was rarely or never used in the management of critical pertussis among PICU physicians.

Other therapeutic approaches were rarely used. The majority (70%) of PICU physicians do not routinely administer corticosteroids to patients with critical pertussis (Table 4). Similarly, 73.5% never or rarely used intravenous immunoglobulins (IVIg). Minority of participants (3.8%) reported using other therapies such as hydroxyurea, bronchodilators, and iloprost. Follow-up post-PICU discharge was available to 8.1% of respondents. Limited access to advanced therapies (including ECMO and high-frequency ventilation) was the most frequently cited barrier to effective management of critical pertussis, followed by limited physician experience, followed by lack of resources. Notably, 86% of physicians believed that having a standardized management guideline for critical pertussis would significantly improve patient care.

**Table 4:** Physicians’ perceptions about the alternate therapies to critical pertussis.

Survey question	*n* (%)
When do you initiate macrolide antibiotics for suspected pertussis?	
Not sure/not answered	1 (0.5)
Upon suspicion	166 (89.7)
After confirmation	18 (9.7)
Do you routinely administer systemic corticosteroids to patients with critical pertussis?	
No	130 (70.2)
Yes, in select cases	51 (27.6)
Yes, routinely	4 (2.2)
How frequently do you use intravenous immunoglobulins (IVIG) for critical pertussis?	
Never	87 (47)
Rarely	49 (26.5)
Occasionally	41 (22.2)
Frequently	8 (4.3)
Have you used any alternative therapies for critical pertussis that were not mentioned above?	
No	178 (96.2)
Yes	7 (3.8)
Does your facility have a follow-up protocol for malignant pertussis survivors post-ICU discharge?	
No	170 (91.9)
Yes	15 (8.1)
What are the barriers to treat critical pertussis in your institution? (Select all that apply)	
Lack of resources	28 (23.7)
Diagnostic delay/ limited availability of test	27 (22.9)
Limited experience with pertussis management	35 (29.7)
Limited access to advanced therapies (e.g., ECMO, HFOV, … etc.)	76 (64.4)
Other barriers (e.g., staff shortages)	8 (6.8)
How would you rate the impact of having a critical pertussis protocol/guideline at your institute on patient outcomes?	
Not sure	3 (1.6)
Very poor	8 (4.3)
Poor	3 (1.6)
Neutral	12 (6.5)
Good	59 (31.9)
Very good	100 (54.1)

CMO, extracorporeal membrane oxygenation; HFOV, high-frequency oscillation ventilation; PICU, pediatric intensive care unit.

Physicians' knowledge of pertussis and its management in the PICU was assessed using a 13-question survey. The average pertussis knowledge score was 9.52 out of 13 questions (SD ±1.72), highlighting a relatively high knowledge on pertussis disease and its management in the PICUs. Considering the percentile analysis, it was found that 25% of the PICU physicians included in the study had scored at least 11 correct points or more. Lowest scored questions were related to pertussis transmission and prevention mainly. Between 47% and 68% of participants were incorrect in identifying the mode of pertussis transmission, risk of outbreaks in vaccinated communities, and timing of antibiotic therapy (Supplementary Table S1).

To better identify and understand predictors of higher pertussis knowledge, a multivariable generalized linear model with gamma distribution was applied to assess factors associated with physicians’ mean pertussis knowledge scores. The analysis (Table 5) revealed that gender, medical specialty, professional designation, healthcare facility type, protocol availability, and PICU capacity were not significantly associated with knowledge scores. In contrast, clinical experience showed a strong and graded association with pertussis knowledge. Compared to physicians with five or fewer years of experience, those with 6–10 years (aRR 1.077, 95% CI: 1.002–1.158, *p* = 0.044), 11–15 years (aRR 1.087, 95% CI: 1.006–1.173, *p* = 0.034), and 16 years or more (aRR 1.114, 95% CI: 1.037–1.199, *p* = 0.004) all had significantly higher knowledge scores. These findings highlight the critical role of cumulative clinical exposure in developing physicians' knowledge and confidence in managing pertussis.

**Table 5:** Multivariable generalized linear regression with gamma for physicians’ Pertussis knowledge score.

Variable	Adjusted Risk Rate	95% CI		*p*-value
		Lower	Upper	
Intercept	2.389	2.151	2.627	<.001
Gender (Male)	1.009	0.958	1.062	0.741
Medical specialty				
Pediatrician	Reference			
Pediatric Intensivist	0.983	0.928	1.041	0.551
Professional designation				
Resident/Registrar	Reference			
Fellow/Senior Registrar	0.926	0.854	1.005	0.065
Consultant/Specialist	0.982	0.918	1.051	0.597
Years of experience				
≤5 years	Reference			
6–10 years	1.077	1.002	1.158	0.044
11–15 years	1.087	1.006	1.173	0.034
≥16 years	1.114	1.037	1.199	0.004
PICU capacity				
Large	Reference			
Medium	1.027	0.971	1.087	0.345
Small	1.007	0.940	1.078	0.852
Number of pertussis cases managed				
>20	Reference			
11–20	0.833	0.658	1.055	0.130
6–10	0.899	0.719	1.123	0.347
3–5	0.843	0.674	1.054	0.135
1–2	0.865	0.690	1.084	0.208
None	0.849	0.669	1.077	0.178
Availability of a hyperleukocytosis protocol				
No	Reference			
Yes	0.969	0.920	1.021	0.235

## Discussion

In this study, we found notable variability in PICU physicians' knowledge, diagnostic access, and management practices for critical pertussis across GCC countries. The findings highlight heterogeneity in clinical decision-making, limitations in access to advanced therapies, and lack of standardized protocols—challenges that collectively may hinder optimal care for infants with life-threatening pertussis. Our findings align with other reports that explore controversies in critical pertussis care, especially on targets for initiation of leukoreduction therapy or ECMO (14, 19, 20).

Our results reveal marked inconsistency in the thresholds used for initiating leukoreduction therapies. While exchange transfusion was the most commonly used modality (60.5%), WBC thresholds for initiating therapy ranged significantly from 30 × 10⁹/L in mechanically ventilated patients to 30–50 × 10⁹/L in less severe cases. This lack of consensus reflects both the paucity of both clinical guidelines and supporting evidence base (16, 21–23). In a recent meta-analysis, the use of exchange transfusion was reported in 12 of 17 included studies with a pooled prevalence of 12% (24).

Despite its use in practice, there is limited evidence to support the routine use of leukoreduction in severe pertussis. A large multicenter prospective cohort found that while extreme leukocytosis was associated with mortality, the use of leukoreduction did not improve survival (16). In contrast, a single-center study that implemented a protocolized leukodepletion strategy in infants with WBC counts ≥50 × 10^9^/L reported improved survival, in comparison to historical cohort (21). These differing outcomes may account for the lack of consensus and guidelines. Notably, nearly 55% of respondents reported the absence of a local protocol for leukoreduction. This gap likely contributes to inconsistent application of interventions and may explain the variability in observed clinical outcomes. In our study, this was evident by the fact that only 54.1% of those who performed leukoreduction reported sustained clinical improvement, and 10.3% reported no clinical changes. Differences in the perceived benefit could be in-prat related to the timing for initiating leukoreduction in relation to symptom onset (25). These findings highlight the pressing need for evidence-based, standardized protocols. Furthermore, integrating data-driven decision-making frameworks, potentially supported by novel artificial intelligence (AI) technologies, could help synthesize clinical, laboratory, and outcome data to guide individualized treatment thresholds and optimize therapeutic strategies in critical pertussis care (26, 27).

While access to mechanical ventilation was nearly universal, ECMO availability was reported by only 24.3% of the respondents, echoing the disparity in critical care infrastructure. Moreover, 36.2% of physicians experienced a delay of two days or more in receiving pertussis PCR test results. Several commercially-available PCR assays have a turnaround time ranging from few hours to more than 24 h depending on several factors such as whether testing is performed in-house or outsourced, laboratory policies regarding test prioritization and batching, and the availability of automated random-access assays; however, this information is often unknown to treating clinicians (28, 29). Timely diagnosis is critical, especially given the rapid progression and high mortality associated with malignant pertussis in early infancy (2). Routine testing for pertussis in PICU patients with respiratory symptoms was infrequent (21.6%), potentially contributing further into delayed diagnosis and missed opportunities for early intervention and infection control, especially during the pertussis surge period. The potential underutilization of echocardiography (performed routinely by only 37.8% of respondents) raises additional critical pertussis management variation, particularly given its value in detecting pulmonary hypertension, one of the key features of malignant pertussis. A consensus guideline recommended routine echocardiography for all infants (20). However, the evidence that guides this practice, including specific patient-level risk factors, timing and frequency of screening echocardiography, is scarce.

The average knowledge score of 9.52 out of 13 (SD ±1.72) indicates a generally good understanding of pertussis among respondents. However, a notable proportion of PICU physicians were unable to correctly identify the primary transmission mode, the risk of outbreaks in vaccinated populations, or the optimal timing for antibiotic therapy. This is concerning given that pertussis outbreaks have been documented even in highly immunized communities, largely due to waning immunity and delayed diagnosis (30). Experience was a significant predictor of knowledge score. Given the rarity of critical pertussis cases, limited exposure may explain the knowledge gaps observed among younger clinicians. Although the number of cases seen in the past 12 months was not a significant predictor of knowledge, cumulative clinical exposure over time may have contributed to these differences. Physicians with more than 5 years of experience outperformed their less experienced counterparts. Although 86% of respondents recognized the value of institutional protocols, fewer than half reported having access to a well-established guideline for critical pertussis care. This gap in structured decision support tools likely contributes to observed inconsistencies in care, as suggested in our results.

This study has several limitations. The study relies on self-reported data, which can potentially introduce recall bias and social desirability biases. Second, our cross-sectional survey design captures perceptions and practices at a single point in time, which may not reflect changes in clinical behavior over time. Furthermore, despite a relatively high number of responses, the use of professional networks for survey distribution may have introduced selection bias, potentially overrepresenting physicians more engaged or interested in pertussis management. In addition, because participation in the survey was voluntary, representation across countries and facility types varied. Moreover, given the cultural, social, and economic similarities among GCC countries, significant sociodemographic variability beyond the country level was not anticipated. Therefore, the primary objective was to assess physicians' knowledge and practices rather than explore associations with demographic variables although limited sociodemographic and professional background data were collected. Nonetheless, this research has important strengths and timely implications. It is the first to comprehensively assess PICU physicians’ knowledge, diagnostic access, and management practices for critical pertussis across all six GCC countries during a period of increased pertussis activity, both regionally and globally. The wide regional representation and inclusion of physicians from a variety of PICU settings, ranging from relatively low-resource to advanced-care units, offer a robust and inclusive understanding of the regional landscape. Furthermore, the study evaluates both diagnostic and therapeutic approaches, including underexplored areas such as thresholds for leukoreduction and perceived barriers to advanced interventions.

## Conclusion

This study reveals significant heterogeneity in the management of critical pertussis across PICUs in the GCC region. Variability in diagnostic access, therapeutic interventions, and clinical decision-making highlights the need for standardized institutional guidelines. Current differences may potentially contribute to inconsistent clinical management and outcomes. Establishing management protocols, improving access to advanced therapies, and harmonizing clinical practice will be essential in improving outcomes for infants with life-threatening pertussis. Enhancing access to advanced therapies, establishing clear protocols, especially around leukoreduction and ECMO, and aligning clinical practices are crucial steps toward improving outcomes in this vulnerable population. Future research should focus on evaluating the effectiveness of specific interventions to inform evidence-based practices. Meanwhile, coordinated efforts to develop regional and international consensus guidelines will be pivotal in advancing the care of infants with life-threatening pertussis.

## Data Availability

The raw data supporting the conclusions of this article will be made available by the authors, without undue reservation.
