# Supplementary material for: Practice variability in the management of critical pertussis: a multicenter survey of pediatric intensivists in the Arabian Gulf Cooperation Council region
Source: Front Pediatr. 2026 Mar 6;14:1662218. doi: 10.3389/fped.2026.1662218 (PMC13002787; doi:10.3389/fped.2026.1662218)
Supplement: Supplementary file 1 [file Table1.docx]

| Supplementary Table.1 Physicians’ knowledge of pertussis presentation, transmission and management. | | |
| --- | --- | --- |
| Knowledge statement | Incorrectly answered  n (%) | Correctly answered  n (%) |
| The primary mode of spread of pertussis is through airborne transmission. | 127 (68.6) | 58 (31.4) |
| The paroxysmal phase of pertussis is characterized by a “whooping” sound following coughing fits. | 25 (13.5) | 160 (86.5) |
| High fever is a common symptom of pertussis infection. | 48 (25.9) | 137 (74.1) |
| The convalescent stage of pertussis can last up to three months. | 26 (14.1) | 159 (85.9) |
| The main goal of antibiotic therapy in pertussis is to reduce the duration of the contagious period. | 18 (9.7) | 167 (90.3) |
| Sedation is sometimes used in the PICU to help manage severe coughing episodes in pertussis cases. | 57 (30.8) | 128 (69.2) |
| Limiting fluid intake is a standard part of supportive care for critically ill patients with pertussis to avoid aspiration. | 45 (24.3) | 140 (75.7) |
| Immunization against pertussis provides lifelong immunity. | 53 (28.6) | 132 (71.4) |
| Pertussis outbreaks can occur in communities with high vaccination rates. | 95 (51.4) | 90 (48.6) |
| The catarrhal stage of pertussis often resembles a common cold. | 4 (2.2) | 181 (97.8) |
| Cyanosis and apnea are more commonly seen in older children with pertussis. | 16 (8.6) | 169 (91.4) |
| Antibiotic treatment is most effective when started during the paroxysmal phase. | 88 (47.6) | 97 (52.4) |
| Prophylactic antibiotics may be given to close contacts of a pertussis case. | 41 (22.2) | 144 (77.8) |
