# Supplementary material for: Practice variability in the management of critical pertussis: a multicenter survey of pediatric intensivists in the Arabian Gulf Cooperation Council region
Source: Front Pediatr. 2026 Mar 6;14:1662218. doi: 10.3389/fped.2026.1662218 (PMC13002787; doi:10.3389/fped.2026.1662218)
Supplement: Supplementary file 2 [file Supplementaryfile1.docx]

Appendix A. Survey Instrument

**GCC Critical Pertussis Research Survey**

1. **Demographics and Background (11 questions)**
2. **What is your medical specialty?**

- Pediatric Intensivist (Fellowship Certified)
- Pediatrician (working in PICU)
- Infectious Diseases Specialist
- Pulmonologist
- General Pediatrician
- Other (Specify ……………………………………)

1. **What is your current professional designation?**

- Consultant/Senior Specialist/Specialist
- Fellow/Senior Registrar
- Resident/Registrar
- Other (Specify ……………………………………)

1. **What is your gender?**

- Male
- Female

1. **In which country is your current healthcare facility located?**

- Bahrain
- Kingdom of Saudi Arabia (KSA)
- Kuwait
- Oman
- Qatar
- United Arab Emirates (UAE)
- Other (Specify ……………………………………)

1. **How many years have you been practicing in your medical specialty?**

- 0-5 years
- 6-10 years
- 11-15 years
- 16+ years

1. **In what type of healthcare facility is it under?**

- Governmental Hospital
- Private Hospital
- University Hospital
- Other (please specify …………………)

1. **What is the classification of your PICU based on bed capacity for non-cardiac ICU beds?**

- Small Capacity PICU (≤ 10 beds)
- Medium Capacity PICU (11–20 beds)
- Large Capacity PICU (21–40 beds)
- Very Large Capacity PICUs (> 40 beds)
- Our medical facility does not have a PICU

1. **How would you describe the resource level of your ICU for managing critically ill children?**

- Basic resources (e.g., High Flow Nasal Cannula (HFNC) &Non-invasive ventilator (NIV), But No Mechanical ventilators)
- Moderate resources (e.g., Mechanical ventilators, No ECMO)
- High-level resources (e.g., ECMO and other advanced critical care technologies)
- Our medical facility does not have a PICU

1. **How many critical pertussis cases admitted to the PICU/HDU have you personally managed in the last 12 months?**

- None
- 1-2 cases
- 3-5 cases
- 6-10 cases
- 11-20 cases
- More than 20 cases

1. **To the best of your knowledge, approximately of all infants (under 12 months old) admitted to your PICU with respiratory symptoms, what percentage are tested for pertussis at your center?**

- 0-10%
- 11-20%
- 21-50%
- Routine test in our PICU for infants admitted with respiratory symptoms
- I am not sure

1. **How frequently do you perform echocardiograms for pertussis cases admitted to your PICU?**

- Always
- In select cases
- Never

1. **Management and Treatment Approaches (12 questions)**
2. **What is your approach to managing hyperleukocytosis in critical pertussis? (select all that apply)**

- Leukapheresis
- Exchange transfusion
- Hyperhydration ( ≥1.5x maintenance fluids )
- Hydroxyurea
- None of the above

1. **What fluid rates do you typically use for hyperleukocytosis in the absence of ARDS?**

- Fluid restriction
- Maintenance
- 1.5x maintenance
- 2x maintenance
- More than double maintenance

1. **In your experience, what is the cutoff WBC level for considering leukoreduction in patients WITHOUT organ dysfunction or oxygen requirement?**

- Will not consider Leukoreduction for such patients
- 30 x 10⁹/L
- 50 x 10⁹/L
- 70 x 10⁹/L
- More than 70 x 10^9^ /L
- I am not sure

1. **In your experience, what is the cutoff point for considering leukoreduction in cases on nasal cannula oxygen ( ≤ 4 L/m)?**

- Will not consider leukoreduction in this case
- 30 x 10^9^ /L
- 50 x 10^9^ /L
- 70 x 10^9^ /L
- More than 70 x 10^9^ /L
- I am not sure

1. **In your experience, what is the cutoff point for leukoreduction in cases requiring non-invasive respiratory support like HFNC, CPAP, or BiPAP?**

- Will not consider leukoreduction in this case
- 30 x 10^9^ /L
- 50 x 10^9^ /L
- 70 x 10^9^ /L
- More than 70 x 10^9^ /L
- I am not sure

1. **In your experience, what is the cutoff point for considering leukoreduction in an intubated patient on a mechanical ventilator?**

- Will not consider leukoreduction in this case
- 30 x 10^9^ /L
- 50 x 10^9^ /L
- 70 x 10^9^ /L
- More than 70 x 10^9^ /L
- I am not sure

1. **Who typically performs leukoreduction procedures at your facility?**

- PICU Team
- Hematology team
- Pediatrician
- Other (Specify …………………………….... )
- I am not sure

1. **From your experience, which leukoreduction methods do you primarily use for managing hyperleukocytosis in pertussis?**

- Plasmapheresis
- Leukapheresis
- Exchange Transfusion
- Cytoreductive medications (e.g., hydroxyurea)
- I have not used leukoreduction strategies
- Other (Specify …………………………………………………)

1. **What challenges do you encounter when using exchange transfusion for critical pertussis? (select all that apply)**

- Resource availability
- Risk of complications
- Lack of experience
- Vascular access
- Uncertainty about its efficacy in pertussis
- Other (Specify …………………………………………….)
- I have never used it before

1. **Which of the following have you considered (in the past) in the management of pulmonary hypertension in malignant pertussis? (select all that apply)**

- Inhaled Nitric Oxide (iNO)
- Sildenafil
- Milrinone
- Heparin
- ECMO
- I have no experience in such a case

1. **How often do you use ECMO in critical pertussis cases?**

- Never used ECMO for pertussis
- 1-5% of the cases
- 6-10% of the cases
- More than 11% of the cases

1. **In your clinical experience, how effective is ECMO in improving the outcomes of malignant pertussis?**

- Highly effective
- Moderately effective
- Minimally effective
- Ineffective
- No experience with such cases

1. **Outcomes, and Institutional Protocols (3 questions)**
2. **What pertussis/clinical outcomes have you observed in patients who underwent Leukoreduction procedures like exchange transfusion or leukapheresis (excluding leukocyte outcomes)?**

- Significant clinical improvement
- Temporary improvement followed by deterioration
- No significant change in outcomes
- Increased complications or worsening condition
- I have not used any leukoreduction procedure in my practice

1. **What complications have you encountered with leukoreduction procedures? (Select all that apply)**

- Hemodynamic instability
- Electrolyte imbalances
- Thrombosis or central venous catheter occlusion/malfunction
- Secondary infection
- Seizures
- Other (Specify ……………………………………………………………..)
- No significant complication

1. **What are the barriers to treating critical pertussis in your institution? (Select all that apply)**

- Lack of resources
- Diagnostic delay/ limited availability of test
- Limited experience with pertussis management
- Limited access to advanced therapies (e.g., ECMO, HFOV, … etc.)
- Other (Specify ………………………………………………….)

1. **Knowledge Test Questions (True/False) – 13 questions**
2. **Pertussis is primarily transmitted through airborne droplets from an infected person.**

- True
- False

1. **The paroxysmal phase of pertussis is characterized by a “whooping” sound following coughing fits.**

- True
- False

1. **High fever is a common symptom of pertussis infection.**

- True
- False

1. **The convalescent stage of pertussis can last up to three months.**

- True
- False

1. **The main goal of antibiotic therapy in pertussis is to reduce the duration of the contagious period.**

- True
- False

1. **Sedation is sometimes used in the PICU to help manage severe coughing episodes in pertussis cases.**

- True
- False

1. **Limiting fluid intake is a standard part of supportive care for critically ill patients with pertussis to avoid aspiration.**

- True
- False

1. **Immunization against pertussis provides lifelong immunity.**

- True
- False

1. **Pertussis outbreaks can occur in communities with high vaccination rates.**

- True
- False

1. **The catarrhal stage of pertussis often resembles a common cold.**

- True
- False

1. **Cyanosis and apnea are more commonly seen in older children with pertussis.**

- True
- False

1. **Antibiotic treatment is most effective when started during the paroxysmal phase.**

- True
- False

1. **Prophylactic antibiotics may be given to close contacts of a pertussis case.**

- True
- False

1. **Diagnosis and Initial Evaluation (3 questions)**
2. **What diagnostic tests are available for pertussis in your center? (Select all that apply)**

- PCR (part of an extended respiratory panel)
- PCR specific to pertussis
- Serology
- Culture
- I am not sure

1. **What is the average turn-around time (time from sample collection to report) for PCR-pertussis testing at your center?**

- Hours (less than 24 hours)
- Days ( 1 – 7 days)
- Weeks (1 – 4 weeks)
- The test is not available

1. **Which of the following symptoms, signs, and laboratory findings trigger you to suspect "Malignant Pertussis"?**

- Hyperleukocytosis
- Rapid clinical deterioration
- Cardiovascular collapse
- Hypoxemia unresponsive to oxygen therapy
- Pulmonary hypertension
- Other (specify ……………………………………………)

1. **Other Management and Treatment (5 questions)**
2. **When do you initiate antibiotic therapy for suspected pertussis?**

- Upon suspicion
- After confirmation
- Only in severe cases

1. **Do you have a protocol for managing hyperleukocytosis in pertussis cases?**

- Yes (well-established)
- Yes (rarely followed)
- No
- Not aware of any

1. **Do you routinely administer systemic corticosteroids to patients with critical pertussis?**

- Yes, routinely
- Yes, in select cases
- No

1. **How frequently do you use intravenous immunoglobulins (IVIG) for critical pertussis?**

- Frequently
- Occasionally
- Rarely
- Never

1. **Have you used any alternative therapies for critical pertussis that were not mentioned above?**

- Yes (Specify ……………………………… )
- No

1. **Follow-up and other suggestions (3 questions)**
2. **Does your facility have a follow-up protocol for malignant pertussis survivors post-ICU discharge?**

- No
- Yes (Specify frequency ………………………………………….)

1. **How would you rate the impact of having a critical pertussis protocol/guideline at your institute on patient outcomes (where 100% represents a “Very Good” impact on a patient’s outcome, 0% represents a “Very Poor” impact on the outcome)?**

- 81 – 100%
- 61 – 80%
- 41 – 60%
- 21 – 40%
- 0 – 20%

1. **Do you have any other comments, questions, or concerns? (Open question)**

- …………………………………………………..…………………………………………
